# Statistical analysis of IMRT dosimetry quality assurance measurements for local delivery guideline

**DOI:** 10.1186/1748-717X-6-27

**Published:** 2011-03-28

**Authors:** Jin Beom Chung, Jae Sung Kim, Sung Whan Ha, Sung-Joon Ye

**Affiliations:** 1 Department of Radiation Oncology, Seoul National University Bundang Hospital Seongnam, Gyeonggi-Do, Korea 463-707; 2 Department of Radiation Oncology and Institute of Radiation Medicine, Seoul National University College of Medicine, Seoul, Korea 110-744; 3 Department of Intelligent Convergence Systems, Graduate School of Convergence Science & Technology, Seoul National University, Seoul, Korea 151-742

## Abstract

**Purpose:**

To establish our institutional guideline for IMRT delivery, we statistically evaluated the results of dosimetry quality assurance (DQA) measurements and derived local confidence limits using the concept confidence limit of |mean|+1.96σ.

**Materials and methods:**

From June 2006 to March 2009, 206 patients with head and neck cancer, prostate cancer, liver cancer, or brain tumor were treated using LINAC-based IMRT technique. In order to determine site specific DQA tolerances at a later stage, a hybrid plan with the same fluence maps as in the treatment plan was generated on CT images of a cylindrical phantom of acryl. Points of measurement using a 0.125 cm^3 ^ion-chamber were typically located in the region of high and uniform doses. The planar dose distributions perpendicular to the central axis were measured by using a diode array in solid water with all fields delivered, and assessed using gamma criteria of 3%/3 mm. The mean values and standard deviations were used to develop the local confidence and tolerance limits. The dose differences and gamma pass rates for the different treatment sites were also evaluated in terms of total monitor uints (MU), MU/cGy, and the number of PTV's pieces.

**Results:**

The mean values and standard deviations of ion-chamber dosimetry differences between calculated and measured doses were -1.6 ± 1.2% for H&N cancer, -0.4 ± 1.2% for prostate and abdominal cancer, and -0.6 ± 1.5% for brain tumor. Most of measured doses (92.2%) agreed with the calculated doses within a tolerance limit of ±3% recommended in the literature. However, we found some systematic under-dosage for all treatment sites. The percentage of points passing the gamma criteria, averaged over all treatment sites was 97.3 ± 3.7%. The gamma pass rate and the agreement of ion-chamber dosimetry generally decreased with increasing the number of PTV's pieces, the degree of modulation (MU/cGy), and the total MU beyond 700. Our local confidence limits were comparable to those of AAPM TG 119 and ESTRO guidelines that were provided as a practical baseline for center-to-center commissioning comparison. Thus, our institutional confidence and action limits for IMRT delivery were set into the same levels of those guidelines.

**Discussion and Conclusions:**

The systematic under-dosage were corrected by tuning up the MLC-related factors (dosimetric gap and transmission) in treatment planning system (TPS) and further by incorporating the tongue-and groove effect into TPS. Institutions that have performed IMRT DQA measurements over a certain period of time need to analyze their accrued DQA data. We confirmed the overall integrity of our IMRT system and established the IMRT delivery guideline during this procedure. Dosimetric corrections for the treatment plans outside of the action level can be suggested only with such rigorous DQA and statistical analysis.

## Introduction

Beamlet-based intensity modulated radiation therapy (IMRT) represents a significant advance in conformal radiation therapy in terms of target dose conformity and normal tissue saving. The dosimetric advantage of IMRT over conventional techniques has been well documented in the literature [1-7]. Due to the inherent complexity in planning and delivery, a comprehensive quality assurance (QA) that ensures the whole process of IMRT should be carried out prior to the treatment [8-11]. Many of the justification, philosophy, and requirements for the IMRT QA program were given in the AAPM and ESTRO guidance document and others [10,12,13]. Patient-specific IMRT quality assurance is one of essential tasks to ensure accurate dose delivery to the patient [14-17]. It often consists of measuring point doses and 2D dose distributions in a phantom. Ion-chambers and 2D arrays of ion-chambers or diodes have been used for this purpose.

Dong et al. [18] extensively analyzed IMRT QA results and found that accuracy in QA of up to ±7% and spatial accuracy of ±5 mm could be achieved. Pawlicki and co-workers [19,20] have reported on the use of control charts for radiotherapy quality assurance of linear accelerators using both hypothetical and clinical data. Breen et al. [21] proposed statistical process control (SPC) concepts for IMRT dosimetric verification. The purpose of SPC was to monitor performance continuously, by testing that the mean and dispersion of the measured data was stable over time. Recently the AAPM TG 119 suggested that the confidence limit for ion-chamber measurements in the target region was 4.5% [13]. For 2D dose comparison, 94% passing rate in gamma criteria [22] of ±3%/3 mm for individual fields and 75% in gamma criteria of ±4%/3 mm for combined fields were proposed in multi-center head and neck IMRT trials [23]. The confidence limit does provide a mechanism for determining reasonable action levels for per-patient IMRT verification studies [13]. The consistency and confidence in advance technology multi-institutional clinical trials was emphasized in the literature [24,25].

We have performed patient-specific IMRT DQA measurements for 206 patients with head and neck (H&N) cancer, brain tumor, abdominal or prostate cancer. Most of point dose measurements (92.2%) agreed with calculated values within ±3%. The average gamma pass rate for criteria of ±3%/3 mm was 97.3 ± 3.7%. However, as reported by international recommendations, treatment planning and delivery in radiation therapy will be never perfect. Thus, we are always engaged to answer the practical question of "how good is good enough or especially what is a reasonable and achievable standard for IMRT commissioning and delivery?" As an institution that has implemented IMRT we statistically analyzed the IMRT DQA results to address this question. Our local values resulted from this study were compared with the tables of AAPM TG 119 report that were provided as a practical baseline for center-to-center comparison. In addition we investigated the agreement of point dose measurements and gamma pass rate for different tumor sites, the number of PTV's pieces, degree of modulation, and the total MU. Based on this rigorous approach, we established our institutional guideline for IMRT delivery.

## Materials and methods

### Planning and Optimization Procedure

From June 2006 to March 2009, 206 patients were treated with IMRT using a Varian Clinac™ 6EX or 21EX Linac equipped with a 120 Millennium™ MLC (Varian Medical Systems, Palo Alto, CA). Patients were grouped into 89 (43.2%) patients with H&N cancer, 42 (20.3%) patients with brain tumor, and 75 (36.4%) patients with abdominal or prostate cancer. IMRT treatment plans consisted of 5 - 11 fields with 6 or 15 MV x-ray beam. Treatment plans were optimized with the Eclipse™ RTP system (version 7.0, Varian, Palo Alto, USA). The optimization process, based on physical constraints in terms of an objective function that describes the limits of acceptance and the goals desired for the dose distribution, created optimized fluence distributions for a number of pre-selected beams, and then transformed them into dynamic MLC movements [26]. The calculation grid used for the final dose distributions was 2.5 mm × 2.5 mm. Especially in H&N cases, the simultaneous integrated boost (SIB) technique was used for concave dose distributions that included the primary tumor and lymph nodes on both sides of the neck [27]. A dose of 2.25 Gy per fraction was delivered to the primary tumor site and involved lymph nodes, and 1.8 Gy per fraction to elective lymph node groups.

### Verification Procedures

Point dose measurements were performed by using a 0.125 cc ion-chamber (semiflex, PTW, Freiburg, Germany) inserted into a cylindrical phantom of acryl. A hybrid plan with the same fluence maps as in the treatment plan was generated on CT images of the phantom in the TPS and delivered to the phantom at the planned gantry and collimator angles. The active volume of the ion-chamber was contoured as a region of interest (ROI) on CT images so that its dose distribution and dose volume histogram were calculated. A point of measurement was selected in the region of high (as high as the prescription dose) and uniform dose distributions, which was usually within the PTV. This was to eliminate issues with very low dose measurements and to minimize uncertainties connected to the MLC movement across the ion-chamber volume and to average the partial volume effect caused by charge integration over the finite volume of chamber [15,16]. The doses of individual fields as well as the sum of them (i.e., total dose) at a point of measurement were recorded, and the difference between calculated (*D_calc_*) and measured doses (*D_meas_*) was calculated as follows:

We paid special attention to any individual field with dose differences larger than 10%. In such cases, another verification plans on CT images of solid water slabs (30 × 30 × 20 cm^3^) were generated to compare calculated and measured 2D dose distributions. All parameters of the treatment plans were identically applied, except for changing the gantry angle to 0 degree. The dose distribution at 5 cm depth from the slab surface was calculated in the TPS and exported for comparison with the measured distribution. Measurements were performed by using a 2D diode array (MapCheck, Sun Nuclear, USA) located at the same 5 cm depth. The evaluation of discrepancy between measured and calculated dose distributions was carried out by using γ-index method [17,22-24] with criteria of 3 mm DTA (distance to agreement) and 3% dose difference. The gamma analysis was restricted to regions to avoid those of very low dose. This was done by defining the region of interest using a threshold dose that was set to 10% of the maximum dose.

## Results

Ion-chamber predictions (i.e., calculated doses) were mean values averaged over the chamber volume segmented on planning CT images. The dose differences between the measured and calculated doses ranged from -4.1% to +3.9% (mean and standard deviation (σ):-0.55 ± 1.51) for the brain case, from -4.6% to +2.7% (-1.62 ± 1.23) for the H&N case, and from -4.6% to +2.5% (-0.41 ± 1.21) for the abdominal or prostate case. The confidence limits for each treatment site were determined by using the concept confidence limit of |mean|+1.96σ. The statistical analysis of point dose measurements for all treatment sites and the resulting confidence limit were shown in Table 1. Our overall local confidence limit was determined to be 0.038 (3.8%), which was better than the value of reference 13 (4.5%). However, in this study under-dosage overwhelmed over-dosage as a ratio of 3.4, and subsequently the mean of percentage dose difference is -0.96% for all cases.

**Table 1:** Statistical analysis of high dose point in the PTV measured with ion-chamber: [(measured dose)-(calculated dose)]/calculated dose, with associated confidence limit

Treatment site	Location	Mean	Standard deviation (σ)	Maximum	Minimum
Prostate	I/C^1 ^or near I/C	-0.004 (-0.4%)	0.012 (1.2%)	0.025 (2.5%)	-0.046 (-4.6%)
Brain	I/C or near I/C	-0.006 (-0.6%)	0.015 (1.5%)	0.039 (3.9%)	-0.041 (-4.1%)
Head and Neck	I/C or near I/C	-0.016 (-1.6%)	0.012 (1.2%)	0.027 (2.7%)	-0.046 (-4.6%)
Overall combined		-0.010 (-1.0%)	0.015 (1.5%)	0.039 (3.9%)	-0.046 (-4.6%)

Confidence limit = (|mean|+1.96σ)			0.038 (3.8%)		

Note: 1. if doses at the isocenter were not fairly uniform, then the ion-chamber was relocated in the region of uniform dose near isocenter. I/C stands for isocenter.

**Figure 1:**
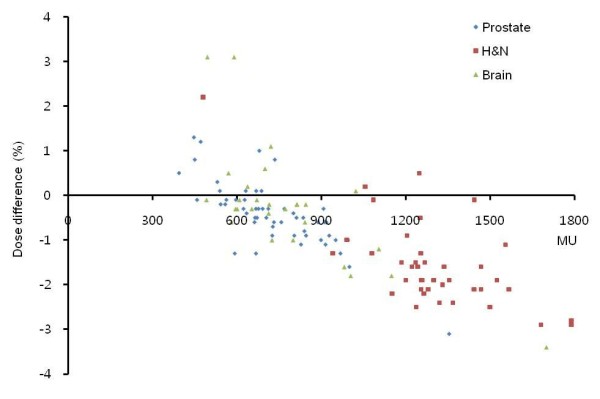
**Distribution of percentage dose differences between measured and calculated doses as a function of total MU for all treatment sites**.

**Figure 2:**
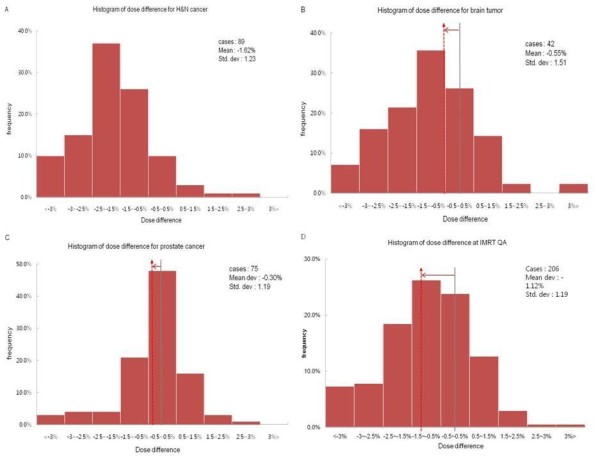
**Frequency histograms vs. percentage dose difference for H&N cancer (a), brain tumor (b), prostate or abdominal cancer (c), and all IMRT cases (d)**. The vertical red dash lines indicate the mean values of percent dose difference for each treatment site with arrows showing the amount of shift from 0%.

**Figure 3:**
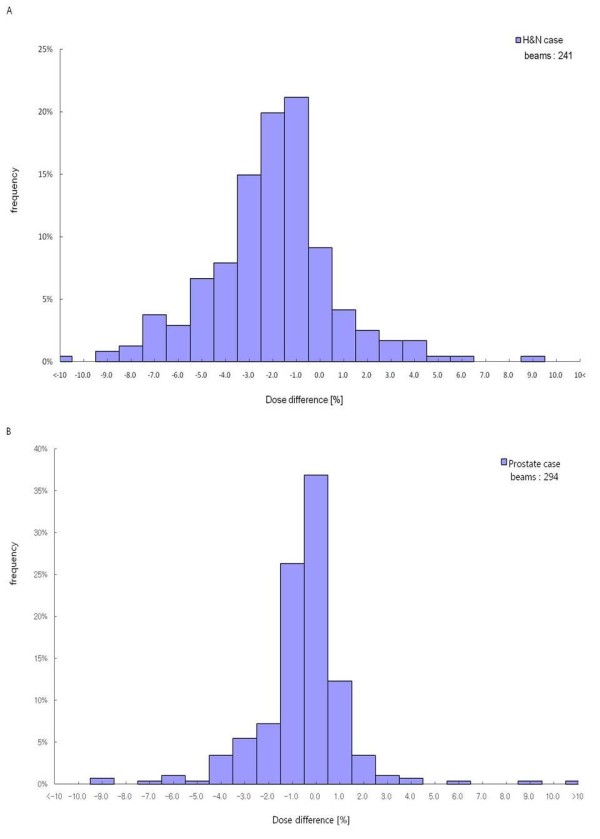
**Frequency histograms vs. percentage dose difference for 241 individual fields of H&N plans (a) and 294 individual fields of prostate or abdominal plans (b)**.

Among 206 DQA results, 16 cases were out of ±3% criteria and only one case was over +3%, while 15 cases below -3%. Figure 1 shows the distribution of dose difference between measured and calculated doses vs. the total MU used for IMRT delivery. The negative trend (i.e., under-dosage) appears beyond the total MU of 700. In figure 1, three symbols illustrate different trends of all three treatment sites. Most of H&N cases were negative with the total MU of > 1,200. Figure 2 shows the frequency histograms of dose difference for three treatment sites and all of them. Even though their mean values are not in the center of -0.5% to 0.5% band, all four histograms seem to be of a Gaussian distribution. A band of -2.5% to -1.5% was most frequent for the H&N case, while a band of -0.5% to 0.5% for the abdominal or prostate case. The tendency of under-dosage (i.e., arrow distance from the center in figure 2) appears to be a systematic error of our IMRT system especially strong for the H&N case, but a random error was a standard deviation (SD) of these Gaussian histograms. Figure 3 shows the frequency histograms of individual field dose differences for the H&N (A) and prostate (B) cases. The IMRT plans of these treatment sites consisted of 8 to 11 fields per plan. The individual field dose differences ranged from -14.4% to11.4%. The frequencies of individual field dose differences larger than ±5% were 12.9% for the H&N case and 3.4% for the prostate case. Some of those large deviations were due to the fact that some fields delivered a very small dose to a point of measurement (typically less than 1-2 cGy), which might be outside of ion-chamber sensitivity.

**Figure 4:**
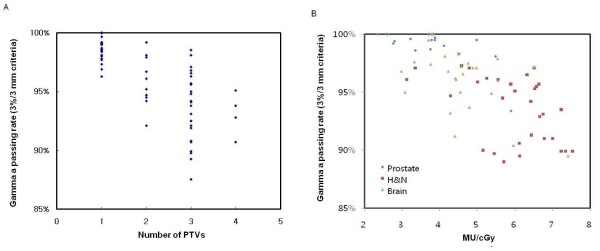
**Gamma (3%/3 mm criteria) passing rates between measured and calculated dose distributions vs. the number of PTV's pieces (a) and MU per cGy (b)**.

We used gamma criteria of 3%/3 mm for 2D dose analysis of composite plans. The percentage of points passing the gamma criteria was on average 95.2 ± 7.4% for the H&N case, 97.1 ± 3.1% for the brain case, and 99.7 ± 0.5% for the abdominal or prostate case. The overall results were 97.3 ± 3.7%. By using the definition described in reference 13, our local confidence limit was determined to be 10.0% (i.e., 90.0% passing). Table 2 summarizes the results of gamma analysis. The failed points were mainly located in the region of high dose gradients or the valley of dose distributions. Some discrepancies between measured and calculated dose distributions seem to be correlated with the number of PTV's pieces and the ratio of total MU to prescription dose (MU/cGy) previously reported in reference 28 [28]. Figure 4 shows the tendencies of gamma passing rate vs. these factors. The pass rate decreases with increasing the number of PTV's pieces and MU/cGy. The mean ratio of total MU to prescription dose averaged over all treatment sites in figure 4(B) was 4.22 MU/cGy. The H&N cases that had the worst pass rate were heavily modulated with an average of 5.96 MU/cGy.

**Table 2:** Percentage of points passing gamma criteria of 3%/3 mm, with associated confidence limits

Treatment site	Location	Mean	Standard deviation (σ)	Maximum	Minimum
Prostate	Isocenter	99.7	0.5	100	92.5
Brain	Isocenter	97.1	3.1	100	89.1
Head and Neck	Isocenter	95.2	7.4	100	82.3
Overall combined		97.3	3.7		

Confidence Limit = (100-mean)+1.96σ			10.0% (i.e., 90% passing)		

## Discussion

The sources of discrepancy between calculated and measured doses are positioning errors of dynamic MLC, insufficient dosimetric data of dynamic MLC in TPS, inaccurate handling of small field dosimetry, and some user's errors and inaccurate measurement devices for setting IMRT QA procedures [29-33]. The accuracy of IMRT DQA results was significantly attributed to dosimetric data of dynamic MLC in TPS such as dosimetric gap, interleaf and mid-leaf leakage, whether tongue-and-groove effect was appropriately handling or not in TPS. The dosimetric gap and leakage of MLC inserted into our TPS were 1.8 mm for 6 MV and 1.875 mm for 15 MV, and 1.6% for 6 MV and 1.8% for 15 MV, respectively, which were consistent with the data used in the other institutions with the same machine and TPS.

The other source of errors in TPS can be the tongue-and-groove effect that often results in under-dosage [34,35]. The tongue-and-groove effect wasn't incorporated into the TPS used in this study. This could more or less contribute to the systematic under-dosage revealed in this study. Recent upgrade of our IMRT system included (1) an advanced dose calculation algorithm that accounts for the tongue-and-groove effect (Eclipse™ 8.6) and (2) the MLC control software (7.2.1 version) that can improve MLC-positioning accuracy. Owing to such improvement, recent results of our DQA measurements don't reveal any noticeable systematic bias. Highly-modulated plans seemed to be more sensitive to accuracies of the above sources of errors than mildly-modulated plans. About 7.8% of highly-modulated H&N plans didn't satisfy ±3% criteria, while the abdominal or prostate IMRT DQA results showed a better agreement (97.3%) than the H&N cases. However, systematic under-dosage was shown in all treatment sites examined in this study. Even considering a large daily dose (225 cGy) of H&N plans compared to a daily dose (200 cGy) of prostate and brain plans, total MU of H&N was much higher that other sites shown in figure 1. This implies the under-dosage trend as increasing the degree of modulation. The complexity of highly-modulated plans most likely exacerbated such a trend of under-dosage. In addition, per-field measurements (see figure 3) showed a broader deviation in H&N than in prostate. This was related to high dose gradients near a point of measurement in highly-modulated H&N plans. With such high dose gradients, a setup error of even less than 1 mm can attribute to a large deviation of per-field measurements.

Recently, IMRT has evolved toward the use of many small radiation fields. Therefore, small ion-chambers with sensitive volumes of ~0.1 cc were often employed for IMRT verification [36,37]. We used an ion-chamber with a 0.125 cc sensitive volume that was the same type of detectors used in most of institutions involved in the AAPM TG 119 study. We believed that the absolute dose error for the IMRT verification measurement within a uniform and high dose region of PTV was minuscule [10].

## Conclusions

Our local confidence limits for both measurements using the ion-chamber and 2D array of diodes were on the same order or less than AAPM TG 119 data [13] and/or ESTRO guidelines [12]. 7.8% of our ion-chamber DQA data were out of ±3% criteria but none of them were on the action level of ±5% recommended in reference 12 and 13. The systematic under-dosage could be corrected by tuning up the MLC-related factors (dosimetric gap, tongue-and-groove effect, and transmission) in the TPS. Institutions that have implemented IMRT in the clinic and performed DQA measurements over a certain period of time need to analyze their DQA data. We confirmed the overall integrity of our IMRT system and established the IMRT delivery guideline during this procedure. Dosimetric corrections for the treatment plans outside of the action levels can be suggested only with such rigorous DQA and statistical analysis.

## Competing Interests

The author(s) declare that they have no competing interests.

## Authors' contributions

Idea and study design: SJY Data collection, analysis and development of methods: JBC, JSK, SWH Manuscript writing: JBC, SJY Manuscript Review and final approval: all authors
